# Genome-Wide Analysis of the LBD Gene Family in Melon and Expression Analysis in Response to Wilt Disease Infection

**DOI:** 10.3390/genes15040442

**Published:** 2024-03-30

**Authors:** Ling Zheng, Yanrong Chao, Yian Wang, Yizhuo Xu, Shipeng Li

**Affiliations:** Department of Biology, Luoyang Normal University, Luoyang 471934, China; cyr200224@163.com (Y.C.); Shipengli@lynu.edu.cn (S.L.)

**Keywords:** melon, LBD family, bioinformatics, expression analysis, wilt disease

## Abstract

LBD transcription factors are a class of transcription factors that regulate the formation of lateral organs, establish boundaries, and control secondary metabolism in plants. In this study, we identified 37 melon LBD transcription factors using bioinformatics methods and analyzed their basic information, chromosomal location, collinearity, evolutionary tree, gene structure, and expression patterns. The results showed that the genes were unevenly distributed across the 13 chromosomes of melon plants, with tandem repeats appearing on chromosomes 11 and 12. These 37 transcription factors can be divided into two major categories, Class I and Class II, and seven subfamilies: Ia, Ib, Ic, Id, Ie, IIa, and IIb. Of the 37 included transcription factors, 25 genes each contained between one to three introns, while the other 12 genes did not contain introns. Through cis-acting element analysis, we identified response elements such as salicylic acid, MeJA, abscisic acid, and auxin, gibberellic acid, as well as light response, stress response, and MYB-specific binding sites. Expression pattern analysis showed that genes in the IIb subfamilies play important roles in the growth and development of various organs in melon plants. Expression analysis found that the majority of melon LBD genes were significantly upregulated after infection with wilt disease, with the strongest response observed in the stem.

## 1. Introduction

The lateral organ boundaries domain (LBD) transcription factor family is widely recognized as a regulatory factor in plant development. LBD originated from the aquatic ancestors of early embryophytes and proliferated in aquatic plants [1]. With the transition of plants from water to land, the LBD family has been preserved and has undergone rapid expansion and functional differentiation, playing an important role in the formation of terrestrial plant characteristics.

LBD is a plant-specific transcription factor family, composed of more than 200 amino acids. These transcription factors have a conserved N-terminus and variable C-termini, with the LOB domain in the N-terminus being the typical feature of this family [2,3]. This domain includes a zinc finger-like structure, GAS region, and leucine zipper-like model. The zinc finger-like structure (CX_2_CX_6_CX_3_C) is composed of four conserved cysteine (C) residues, interspersed with some non-conserved amino acid residues (X), which is related to binding with downstream genes [3]. The GAS region is composed of glycine, alanine, and serine, which can assist the zinc finger-like structure in binding with the gene promoter [3]. Furthermore, the C-terminus of the GAS region has a conserved proline residue (Pro) that affects the biological function of the LBD gene and its binding activity with DNA [4]. The leucine zipper-like model (LX_6_LX_3_LX_6_L) is composed of four conserved leucine residues (L), which are mainly responsible for protein dimerization [2,3].

The LBD gene family of numerous plants has been identified on a whole-genome scale. In *Arabidopsis*, the first discovery accounted for 43 LBD genes [3]. Additionally, 35 and 131 LBD genes were identified in rice and upland cotton, respectively [5,6]. Within the Rosaceae family, 58 and 37 LBD genes were identified in apple and strawberry, respectively [7,8]. LBD transcription factors encompass various biological functions. For instance, *AtLOB*, the first factor isolated from *Arabidopsis*, regulates early leaf development through interacting with SHOOT MERISTEMLESS and BREVIPEDI CELLUS proteins [2]. In *Arabidopsis*, the three N/NO(3)(-) induced members, *LBD37*, *LBD38*, and *LBD39*, serve as negative regulators of anthocyanin biosynthesis, while the rice gene *Os-LBD37/ASL39* participates in nitrogen metabolism [9,10]. In sugar beet, it has been discovered that the interaction between AUX and BNYVV proteins leads to high expression of *LBD16*, *LBD18*, and *LBD29* during the disease phase, subsequently causing a surge in lateral root growth and a decrease in sugar beet yield [11,12]. In the scenario of grapes under salt stress and low-temperature treatment, the expression of genes *LBD4* and *LBD18* notably decreases. Conversely, under mannitol and heat stress conditions, the expression of *LBD4* and *LBD18* significantly increases [13].

Melon (*Cucumis melo* L.), also known as cantaloupe or honeydew, is among the earliest melon fruits consumed in China and has substantial economic value. *Fusarium* wilt, a disease caused by the fungus *Fusarium oxysporum*, is one of the most common diseases affecting melons [14]. As of now, there is no research on the response of the melon LBD gene family to *Fusarium* wilt stress. This paper mainly deciphers the basic information of this transcription factor family through whole-genome phylogenetic analysis, chromosomal localization analysis, gene structure analysis, expression profile analysis, cis-acting element analysis, and expression analysis post *Fusarium* inoculation. This information provides insights into the mechanism of its role in the growth and development of melons. The findings serve as a basis for promoting functional research of the melon LBD gene and molecular breeding.

## 2. Materials and Methods

### 2.1. Identification of the Melon LBD Gene Family

Amino acid sequences of the LBD family from *C. melo* and *Arabidopsis thaliana* were acquired from the Plant Transcription Factor Database (PlantTFDB, http://planttfdb.gao-lab.org/, accessed on 20 February 2022). Using the Hidden Markov Model (HMM) file for the LOB domain (PF03159) from the Pfam protein family database (http://pfam.xfam.org/, accessed on 20 February 2022) as a seed model, predicted LBD proteins were searched in the *C. melo* genome database (http://cucurbitgenomics.org/, accessed on 20 February 2022) with an E-value of 1 × 10^−10^. Subsequent alignment and removal of duplicate sequences facilitated the identification of candidate genes. Candidate genes were then validated online using InterPro (http://www.ebi.ac.uk/interpro/) (accessed on 20 February 2022), conclusively determining the members of the melon LBD family. The final candidate sequences were analyzed using ExPASy (http://www.expasy.org/) (accessed on 20 February 2022) to determine various physicochemical properties of the melon LBD gene family proteins. The subcellular localization of the sequences was determined using the Plant-Ploc (http://www.csbio.sjtu.edu.cn/bioinf/plant/?tdsourcetag=s_pcqq_aiomsg, accessed on 20 February 2022), and the data were recorded and tabulated.

### 2.2. Chromosomal Position Analysis of Melon LBD Gene Family

The CmLBD family genes were searched in the Cucurbit Genomics Database, yielding the chromosomal location information of 37 genes. The information for each chromosome was converted into corresponding files, and the chromosomal location map was created using the MapInspect 1.0.

### 2.3. Construction of the Phylogenetic Tree for the Melon LBD Gene Family

The phylogenetic tree of the LBD gene family of *A. thaliana* and melon were constructed using MEGA 7.0 software. MUSCLE multiple sequence alignment was performed. We searched for the best model using MEGA 7.0 and constructed the neighbor-joining (NJ) phylogenetic tree. After a bootstrap test of 1000 replicates, the model of JTT + G was selected as the best model, and pairwise deletion.

### 2.4. Synteny Analysis of Melon LBD Gene Family

The downloaded melon protein sequences were aligned on TBtools to produce a file describing the similarities between the melon genes. This file was then compared with the melon LBD gene file to produce a synteny analysis diagram.

### 2.5. Gene Structure Analysis of the Melon LBD Family

Utilizing the Cucurbit Genomics Database, melon genomic information was retrieved, followed by the sequential extraction of corresponding CDS and GENE sequences. The analysis was conducted using the GSDS 2.0 (http://gsds.gao-lab.org/index.php, accessed on 25 February 2022), facilitating the generation of exon-intron structure diagrams for the *C. melo* LBD gene family. The identification of conserved motifs within the melon LBD gene family was performed using the online tool MEME (http://meme-suite.org/tools/meme, accessed on 25 February 2022), with the number of conserved motifs set to 10. The obtained results were further analyzed through the Redraw Motif Pattern tool in TBtools, culminating in the visualization of the motif patterns.

### 2.6. Expression Pattern Analysis of Melon LBD Gene Family

The Melonet DB (https://melonet-db.dna.affrc.go.jp/ap/top, accessed on 25 February 2022) was used to retrieve the expression data of 37 LBD genes in different organs and at different time points in plant development based on the melon gene sequence numbers. The TBtools software was then used to create a heatmap of gene expression.

### 2.7. Promoter Analysis of the Melon LBD Gene Family

An information file on CmLBD was obtained through operations in the TBtools. This file was then used to make predictions via the PlantCARE tool (http://bioinformatics.psb.ugent.be/webtools/plantcare/html/, accessed on 20 February 2022) yielding a TAB file containing cis-acting element-related data, from which redundant values were manually removed. The cis-acting elements diagram was then generated using the GSDS2.0 website.

### 2.8. RNA Extraction, cDNA Synthesis, and qRT-PCR of the Melon LBD Gene Family

Seeds of the melon cultivar “Super Sweet White Sugar Jar” were soaked, germinated, and sown. The plants were grown until the three-leaf stage in a greenhouse under 16/8 h (day/night) light exposure and temperatures of 28/18 °C (day/night). *F. oxysporum* was isolated from diseased melon plants. A suspension containing 1 × 10^8^ spores/mL was prepared, and the plants were inoculated with 10 mL each via root drenching. At 0, 24, 48, and 96 h post-inoculation, the roots, mid-stem, and first true leaf were harvested and immediately frozen in liquid nitrogen for storage at −80 °C, with three biological replicates per treatment.

To investigate the spatiotemporal expression patterns of the LBD family under *Fusarium* wilt infection, we utilized a total of 12 tissues obtained from four key growth stages of the melon. RNA extraction was performed, and the RNA quality and concentration were evaluated using Nanodrop 2000. First strand cDNA was synthesized using SweScript All-in-One First-Strand cDNA Synthesis (TRANS, G3337). qPCR was performed using the 2 × SYBR Green qPCR Master Mix (None ROX) (TRANS, G3320) in a CFX96 real-time PCR detection system (Bio-Rad, Hercules, CA). The cycling conditions were as follows: 95 °C for 30 s, followed by 40 cycles of 95 °C for 15 s, and then 60 °C for 30 s. The actin (CuGenDB name: MELO3C008032) gene was used as the internal control for normalization. Three biological replicates with three technical replicates were assayed for each sample. Reactions for the reference gene were included in each plate. The mRNA relative expression levels of LBD family genes were calculated with the formula 2^−ΔΔCT^. The data was compiled using SPSS.16.0 and the differences between treatments (24, 48, and 96 h plant tissue) and the control (0 h plant tissue) were evaluated using one-way analysis of variance (ANOVA) followed by a least significant difference (LSD) test. Statistical significance and high significance were indicated as ** *p* < 0.01 and * *p* < 0.05, respectively.

### 2.9. Protein-Protein Interaction Network of CmLBDs

The LBD protein sequences were uploaded to the STRING database (https://string-db.org/, accessed on 8 March 2024) for node alignment, and relationships among key proteins were predicted based on protein-protein interactions in *A. thaliana*. Cytoscape (V3.7.1) was utilized to visualize the generated network.

## 3. Results

### 3.1. Identification and Analysis of Melon LBD Gene Family

Through the Plant TFDB, CuGenDB, 37 LBD transcription factors were identified. As shown in Table 1, the protein length of CmLBD factors ranges from 96 (CmLBD13)~310 (CmLBD25) aa. The protein molecular weight ranges from 10,508.77 Da (CmLBD13) to 34,948.92 Da (CmLBD25). The isoelectric point ranges from 4.45 (CmLBD31) to 9.43 (CmLBD34), with 24 greater than seven (alkaline) and 13 less than seven (acidic). Among the 37 melon LBD proteins, only two is stable, with an instability index less than 40 (CmLBD13 and CmLBD15), while the remaining 35 LBD proteins are unstable, with instability indexes greater than 40. Only CmLBD1,CmLBD17 and CmLBD21 are hydrophilic proteins with positive average hydrophilicity, while the rest are hydrophobic proteins. Subcellular localization analysis showed that all 37 melon LBD genes are located in the cell nucleus.

### 3.2. Chromosomal Distribution and Synteny Analysis of Melon LBD Gene Family

**Figure 1:**
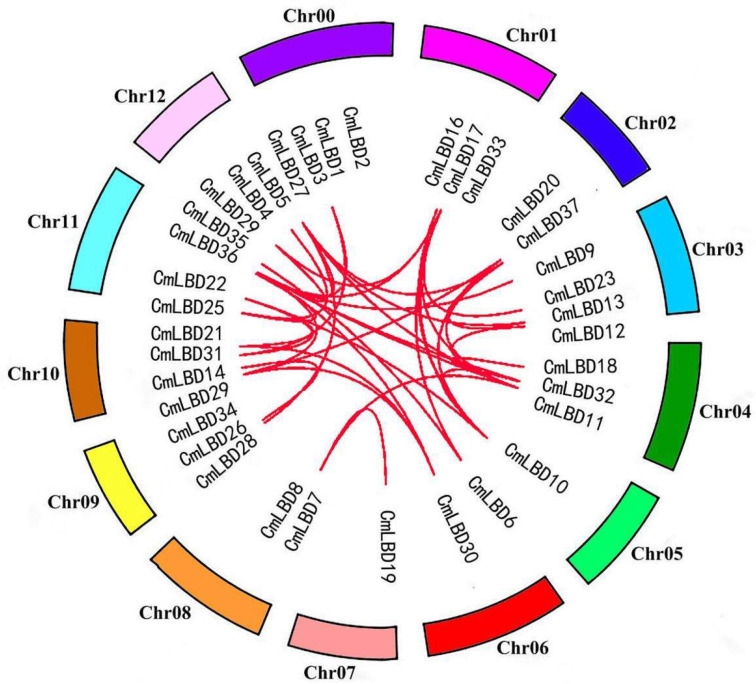
Collinear analysis of LBD family genes in melon.

**Figure 2:**
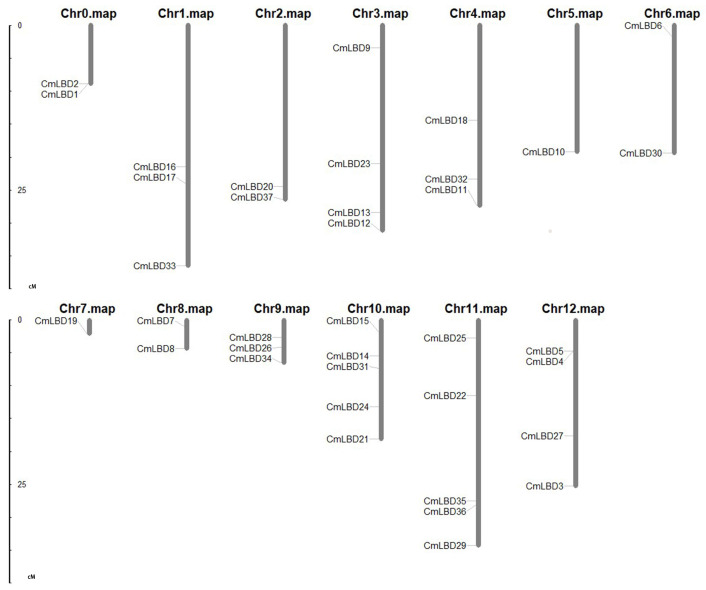
The location distribution of LBD genes in melon chromosomes.

The synteny analysis diagram (Figure 1) and chromosomal location diagram (Figure 2) of the melon LBD gene family were plotted respectively. The LBD genes are unevenly distributed across 13 chromosomes, with chromosome 10 and 11 having the highest number of genes (five), and chromosomes 5, 7 having the least (one each). Only two genes are distributed on chromosomes 0, 2, 6, and 8. Homologous genes on a chromosome can be considered tandem repeat genes if their physical positions do not exceed 100 kb [15]. A tandem duplication event (*CmLBD1/2*) was identified in the melon genome. Additionally, there are 38 segmental duplication gene pairs present within the duplicated segments of the melon genome. These findings suggest that some CmLBDs may have originated from gene duplication, with segmental duplication events being a major driving force in the evolution of CmLBDs. Gene duplication events are common in all species, as they can generate new functional genes and drive species evolution [16,17].

### 3.3. Construction of the Phylogenetic Tree of Melon and Arabidopsis LBD Gene Family Members

**Figure 3:**
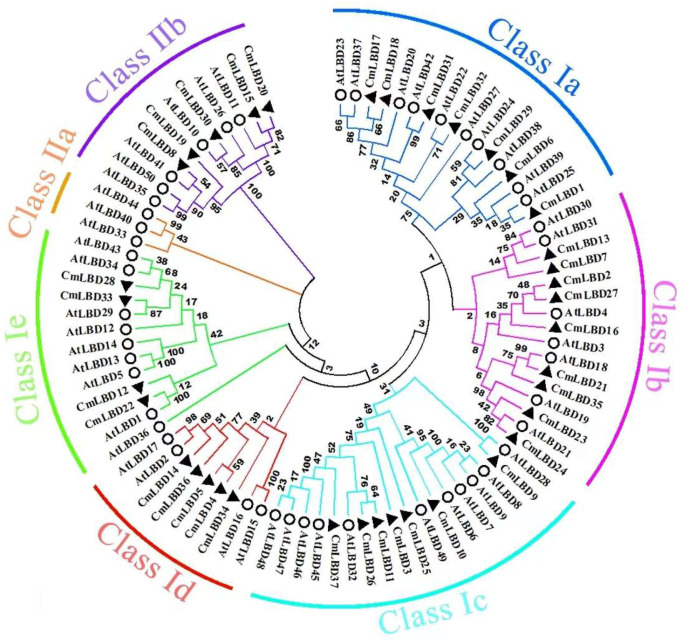
The neighbor-joining (NJ) phylogenetic tree of the CmLBD gene family.

Based on the classification of the LBD family in *Arabidopsis*, the melon LBD proteins can be divided into two major classes, I and II. Class I contains five subclasses (Ia, Ib, Ic, Id, Ie), and Class II contains two subclasses (IIa, IIb) (Figure 3). Six subclasses contain members from both species, and members within each subclass may exhibit some degree of structural and functional similarity. The distribution of melon LBD genes in these seven subclasses is uneven, with the most members (nine) in Ib. There are no members of CmLBD in IIa. The neighbor-joining (NJ) phylogenetic tree analysis of melon and *Arabidopsis* reveals that there are five pairs of orthologous paralog genes in the melon LBD family: *CmLBD4* and *CmLBD5*, *CmLBD15* and *CmLBD20*, *CmLBD11* and *CmLBD26*, *CmLBD2* and *CmLBD27*, and *CmLBD17* and *CmLBD18.*

### 3.4. Conservation Domain Analysis of Melon LBD Family Genes

**Figure 4:**
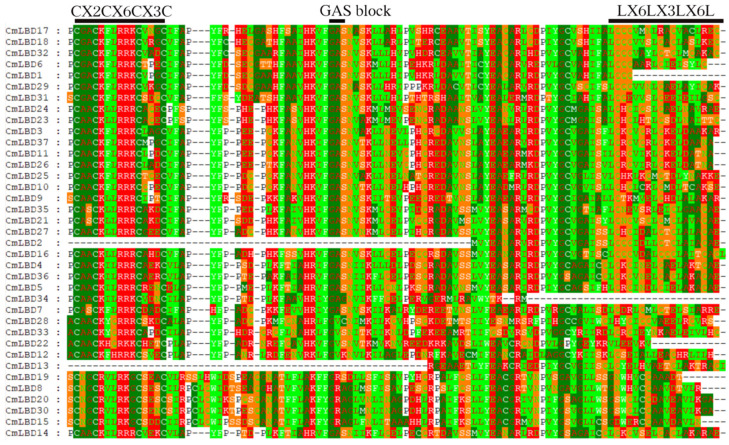
Conserved domain analysis of LBD family genes in melon.

We performed an alignment of the protein sequences of the 37 members of the melon LBD gene family (Figure 4). The LOB domain is mainly composed of three conserved sequences: the zinc finger-like structure (CX_2_CX_6_CX_3_C), the GAS region, and the leucine-zipper-like model (LX_6_LX_3_LX_6_L). Among these, CmLBD2 and CmLBD13 completely lack both the zinc finger-like structure and the GAS region. CmLBD34 completely lacks the leucine-zipper-like model, while CmLBD1, CmLBD19, and CmLBD22 have partially truncated leucine-zipper-like models. The leucine-zipper-like model has relatively more mutations compared to the other two conserved sequences.

### 3.5. Gene Structure and Conserved Motif Analysis of Melon LBD Family Genes

**Figure 5:**
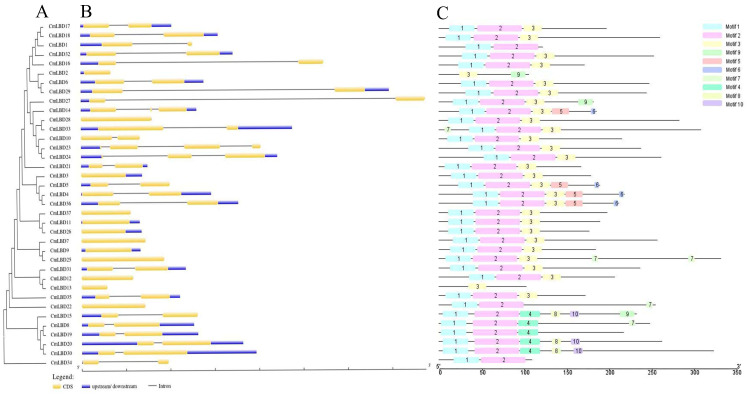
Gene structure and phylogeny of the melon LBD family. (**A**) The neighbor-joining (NJ) phylogenetic tree for the CmLBD protein family. (**B**) Exon–intron structure of CmLBD genes. (**C**) Distribution of 10 motifs in 37 CmLBD proteins.

The number of introns in the 37 LBD genes ranges from one to three (Figure 5B). Twelve of these genes lack introns; two genes (*CmLBD14*, *CmLBD24*) contain two introns, and only one gene (*CmLBD23*) contains three introns. The remaining genes all contain one intron. Genes within the same subfamily generally have similar gene structures with minor differences. For example, in Class IIb, all five genes each contain one intron.

Using the MEME website to analyze the melon LBD family protein sequences, a total of 10 conserved motifs were identified (Figure 5C). As can be seen in Figure 5, while *CmLBD2* and *CmLBD13* do not contain motifs 1 and 2, all of the other CmLBDs do, indicating a high degree of conservation in CmLBD. Motif 3 also appears frequently, present in 29 members, but motif 9 is only found in two transcription factors (*CmLBD2* and *CmLBD27*). Some motifs only appear in specific subfamilies, such as motifs 8 and 10, which are only found in the IIb subfamily. Research on the melon LBD gene family reveals that genes within the same subfamily are similar in terms of gene structure and conserved sequences. This suggests that they likely have similar functions.

### 3.6. Expression Pattern Analysis of Melon LBD Family Genes

**Figure 6:**
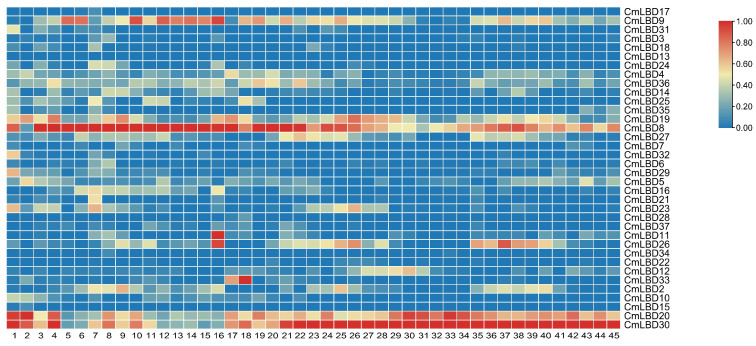
Expression analysis of LBD genes in melon. 1: callus, 2: dry seeds, 3: 1d-imbibed seeds, 4: 3d-imbibed seeds, 5: 7d-seedlings cotyledon, 6: 7d-seedlings hypocotyl, 7: 7d-seedlings root, 8: root, 9: middle stem, 10: upside stem, 11: shoot apex, 12: young leaves, 13: 6th leaves, 14: 9th leaves, 15: 12th leaves, 16: tendril, 17: anther male flower, 18: anther female flower, 19: petal female flower, 20: stigma female flower, 21: ovary DAF0, 22: ovary DAF2, 23: ovary DAF4, 24: fruit flesh DAF8, 25: fruit flesh DAF15, 26: fruit flesh DAF22, 27: fruit flesh DAF29, 28: fruit flesh DAF36, 29: fruit flesh DAF43, 30: fruit flesh DAF50, 31: fruit flesh post harvest 1 week, 32: fruit flesh post harvest 2 week, 33: fruit flesh post harvest 3 week, 34: fruit flesh post harvest 4 week, 35: fruit epicarp DAF8, 36: fruit epicarp DAF15, 37: fruit epicarp DAF22, 38: fruit epicarp DAF29, 39: fruit epicarp DAF36, 40: fruit epicarp DAF43, 41: fruit epicarp DAF50, 42: fruit epicarp post harvest 1 week, 43: fruit epicarp post harvest 2 week, 44: fruit epicarp post harvest 3 week, 45: fruit epicarp post harvest 4 week.

To further explore the function of LBD genes, we retrieved the expression specifics of melon LBD family genes in different tissues and at different time points through the Melonet DB website (Figure 6). The results showed that, among the 37 genes retrieved, only *CmLBD13* was not expressed. The remaining genes showed significant differences in expression levels in different organs and at different times, demonstrating tissue specificity.

Genes such as *CmLBD24*, *CmLBD8*, *CmLBD23*, *CmLBD16*, *CmLBD20*, and *CmLBD30* have higher expression levels in roots, suggesting that they may be involved in root development and affect root function. *CmLBD9*, *CmLBD19*, *CmLBD8*, and *CmLBD2* show higher expression in stems. *CmLBD9*, *CmLBD36*, and *CmLBD8* have higher expression levels in leaves. *CmLBD9*, *CmLBD36*, *CmLBD8*, *CmLBD20*, and *CmLBD30* show significant expression in flowers, indicating that they may promote the formation and development of melon flowers. *CmLBD9*, *CmLBD19*, *CmLBD8*, *CmLBD12*, *CmLBD20*, and *CmLBD30* have high expression levels in fruits. In seeds, the highest expression levels are seen in *CmLBD9*, *CmLBD5*, *CmLBD20*, and *CmLBD30*, suggesting they may promote the formation of melon seeds.

Expression analysis (Figure 6) showed that *CmLBD8*, *CmLBD20*, and *CmLBD30*, which are all from subfamily IIb, have higher expression levels in roots, stems, leaves, flowers, fruits, and seeds. We speculate that genes from subfamily IIb participate in the development of various organs in melons.

### 3.7. Cis-Acting Element Analysis of Melon LBD Family Genes

**Figure 7:**
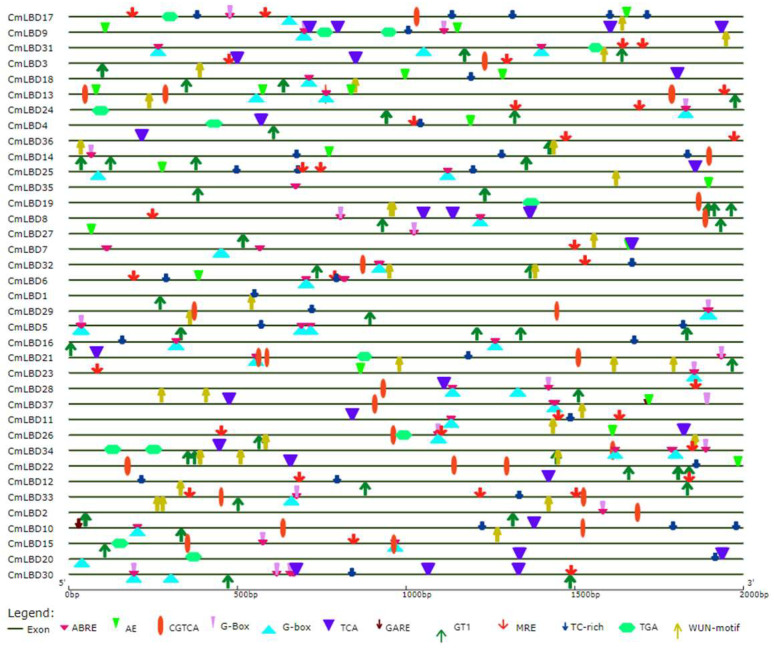
Cis-elements of LBD genes in melon.

Cis-acting elements are non-coding DNA sequences in the gene promoter that regulate the transcription of related genes [18]. In melon LBD genes, a total of 12 cis-acting elements were identified, categorized into four classes: plant hormone response, light response, stress response, and specific binding sites for MYB (Figure 7). (1) Elements related to plant hormone responses were the most numerous, including abscisic acid-responsive elements (ABRE), methyl jasmonate(MeJA) responsiveness, salicylic acid-responsive elements (TCA), auxin-responsive elements (TGA), and gibberellin-responsive element (GARE). (2) The light response category includes light-responsive elements (G-box), a part of a module for light response (AE box), and light-responsive elements (GT1). (3) The stress response class, involved in hypoxia-specific induction, includes two elements, namely the defense and stress response elements (TC-rich), and wound response elements (WUN-motif). (4) MYB binding sites (MRE) are involved in light responses.

Among the five types of hormone response elements, ABRE has the widest distribution, across 56 sites, while the GARE-motif has the least distribution, across only two sites. ABA-responsive elements (ABRE) were found in most genes, such as *CmLBD31*, *CmLBD7*, *CmLBD5*, *CmLBD34*, *CmLBD33*, and *CmLBD30*, and were distributed across all subfamilies. Light-responsive elements (GT1), MYB binding sites involved in light response (MRE), and wound response elements (WUN) were also found in multiple CmLBD genes, and these three response elements are distributed across all subfamilies.

### 3.8. Expression of Melon LBD Family Genes in Response to Wilt Disease Pathogen

**Figure 8:**
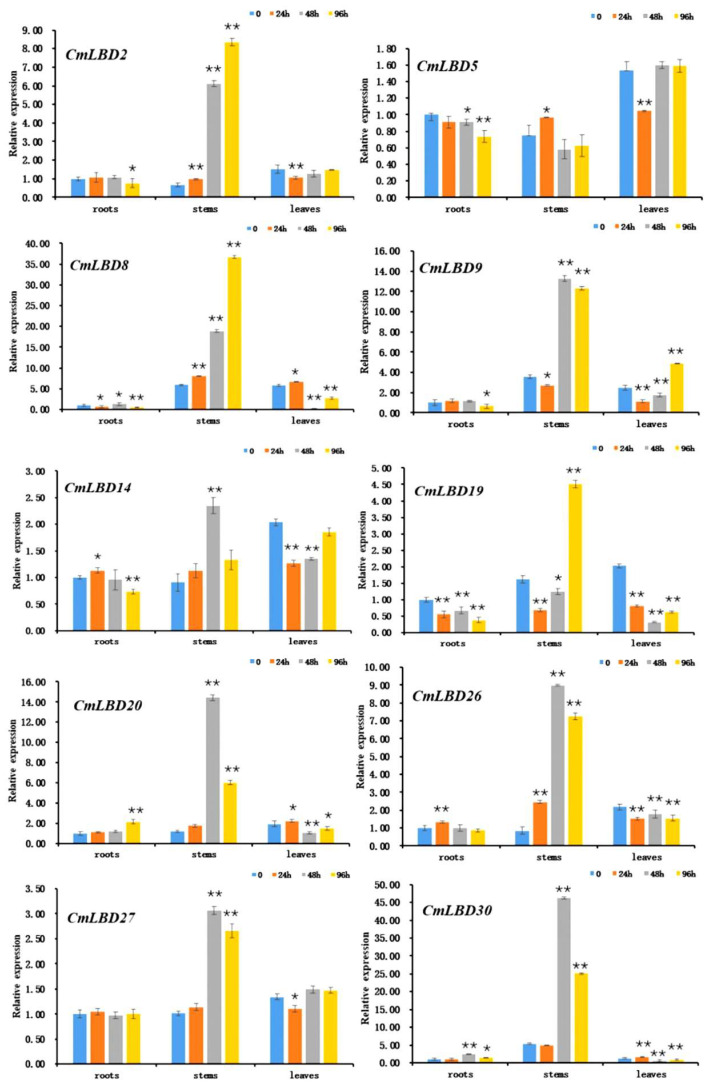
Expression of melon LBD family genes in response to wilt bacteria. The asterisk indicates the *p* value in the significance test (** *p* < 0.01 and * *p* < 0.05).

The response of melon LBD family genes to wilt disease pathogen stress was studied using qRT-PCR. The results showed (Figure 8) that 10 LBD family genes exhibited high expression in roots, stems, and leaves after inoculation with *F. oxysporum*. Most of these genes had significantly upregulated expression levels in stems (*CmLBD2*, *CmLBD8*, *CmLBD9*, *CmLBD14*, *CmLBD19*, *CmLBD20*, *CmLBD26*, *CmLBD27*, *CmLBD30*), especially at 48 h and 96 h post-inoculation, when expression levels increased by several to tens of times. Moreover, most genes showed a trend of initial increase and subsequent decrease, reaching peak expression at 48 h post-inoculation, with a few genes exhibiting a continuous increase and reaching maximum values at 96 h post-inoculation (*CmLBD2*, *CmLBD8*, *CmLBD19*).

Four LBD family genes had a certain degree of downregulation in roots (*CmLBD2*, *CmLBD8*, *CmLBD9*, *CmLBD19*), whereas *CmLBD20* had a certain degree of upregulation. The expression of *CmLBD8*, *CmLBD19*, *CmLBD20*, and *CmLBD30* in leaves significantly decreased with increased inoculation time, whereas *CmLBD9* exhibited significant upregulation. The genes of Class II (*CmLBD20*, *CmLBD30*) responded strongly in the stems after inoculation, and also showed a certain degree of up- and downregulation in roots and leaves.

### 3.9. Protein Interactions

**Figure 9:**
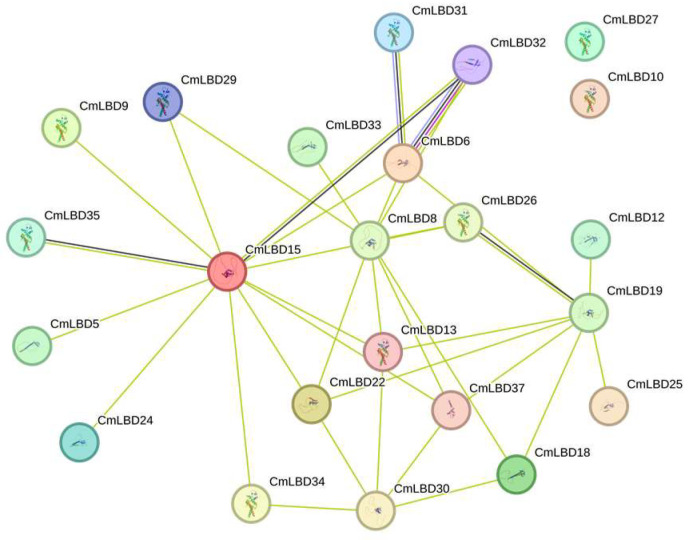
*C. melo* CmLBD functional interaction networks based on Arabidopsis orthologs.

Potential interactions among CmLBD proteins were predicted using the STRING database (Figure 9). The CmLBD protein interaction network consists of 21 nodes, each communicating with other nodes. It was observed that *CmLBD15* acts as a central hub in the network with numerous connections, suggesting its potential core role in regulating the network. In contrast, nodes like *CmLBD5*, despite having fewer connections, may still participate in specific interactions. Complex multigene interaction relationships, such as the connections between *CmLBD6*, *CmLBD8*, and *CmLBD26*, might indicate a synergistic function of these proteins within the network. This network diagram can assist in predicting the complexity of gene regulation and their potential roles in biological processes.

## 4. Discussion

LBD transcription factors are plant-specific transcription factors that play crucial roles in plant growth, development, and responses to abiotic stress. So far, 43, 35, and 44 members of the LBD transcription factor family have been identified in *Arabidopsis*, rice, and maize, respectively [3,5,19]. Using bioinformatics methods, this study identified 37 LBD genes in melon, unevenly distributed across 13 chromosomes. Only one LBD gene each was found on chromosomes 5 and 7, while the highest number of genes, five, was found on chromosome 10 and 11. Subcellular localization of all 37 genes was determined using the Plant-Ploc, and all were found to be located in the nucleus. Upon analyzing the structure of the LBD gene family, it was found that 32% of the genes did not contain introns, while 68% contained one to three introns. In addition, it was found that all genes except CmLBD2 and CmLBD13 contained motif 1 and motif 2. Thus, it can be seen that most LBD genes have a similar exon-intron structure and the same conserved elements, indicating that the LBD family is widely conserved in the evolutionary process.

Research has shown that the majority of LBD proteins belong to Class I. A total of 43 LBD transcription factors have been identified in the model plant *Arabidopsis*, 37 of which belong to Class I, and 6 to Class II. In *Physcomitrella patens*, a total of 31 LBD transcription factors have been identified, including 24 Class I members and 7 Class II members [20]. Among the 37 LBD genes identified in melon, 32 belong to Class I and 5 belong to Class II (Figure 3), further corroborating that the number of Class I members in the LBD gene family significantly outnumbers Class II members across different species.

Phylogenetic tree analysis indicates that each subfamily contains related genes from both melon and *Arabidopsis*, suggesting these genes share a common ancestral origin. The constructed phylogenetic tree reveals a close evolutionary relationship between melon and *Arabidopsis*, indicating the CmLBD genes are highly conserved throughout evolution [16]. The expression levels of genes located on the same branch are similar (e.g., *CmLBD8* and *CmLBD19*, *CmLBD20* and *CmLBD30*, *CmLBD4* and *CmLBD36*) (Figure 3 and Figure 6), suggesting that duplication of LBD genes may primarily lead to functional redundancy. Exon/intron structure analysis the vast majority of CmLBDs contain no more than two introns, which is largely consistent with LBD genes in other plants, indicating a relatively conservative gene structure throughout evolution [21]. Gene function is closely related to the presence of conserved motifs within the protein sequence [22]. The expansion of the CmLBD gene family may primarily occur through segmental duplication, as 38 pairs of segmentally duplicated CmLBD genes were identified, while only one tandemly duplicated CmLBD gene pair (*CmLBD1/2*) was found, similar to other species from different taxonomic groups [21,23]. We hypothesize that during evolution, CmLBD gene family amplification was dominated by a segmental replication mechanism and supplemented by a tandem replication mechanism. This contributes to the development of new gene functions and may explain the relatively conservative number of CmLBD gene family members. Conservative motif analysis revealed that CmLBD proteins belonging to the same cluster possess similar conserved motifs. Proteins appearing in the same cluster are likely to have similar functions. Compared to other transcription factors, *CmLBD8*, *CmLBD19*, *CmLBD20*, and *CmLBD30* not only share motif 1 and motif 2, but also uniquely possess motif 4. This finding implies these genes may play similar roles in plant physiological functions. Moreover, gene structure analysis reveals that these genes all have a single intron. These structural characteristics might reflect specific adaptations during evolution, thereby maintaining functional consistency. These genes exhibit a strong expression pattern in roots, stems, and leaves after inoculation with *F. oxysporum*, suggesting that motif 4 might be closely related to the function of the LBD family in resisting biotic stress. Further, we note that members of the subfamily IIb, such as *CmLBD20* and *CmLBD30*, besides having motif 1, motif 2, and motif 4, uniquely contain motif 8 and motif 10. The high expression of these two genes in flowers, fruits, and seeds draws our attention, suggesting that motif 8 and motif 10 may be closely related to reproductive traits in melon, such as flower development, fruit maturation, and seed formation. These findings provide important insights into the diverse functions of LBD family members in plant growth, development, and response to environmental stresses.

In the LBD gene family, Class I primarily regulates growth and development, while Class II may be associated with environmental stress responses [2,16]. Our research reveals that several genes within Class II, including *CmLBD8*, *CmLBD20*, and *CmLBD30*, not only play significant roles in response to biotic stress, but also in the development of crucial organs such as flowers, fruits, and seeds in melon (Figure 6 and Figure 8). This enhances our understanding of the functions of the LBD family’s Class II category. Phylogenetic analysis of melon LBD proteins and those of *Arabidopsis* suggests that LBD genes are more evolutionarily recent, and those that cluster together in the same major class or subclass likely have similar structures and functions [24]. In *Arabidopsis*, *AtLBD6* can inhibit the division and differentiation of cells near the mid-axial region, resulting in the formation of bilaterally symmetrical, planar leaves [25]. Thus, it is hypothesized that *CmLBD10*, which is homologous to *AtLBD6*, may have a similar function. *AtLBD12* is involved in the development of the leaf [26], suggesting that CmLBD genes, which belong to the same branch, may regulate similar function.

The tissue expression patterns of genes are closely related to their functional features. In this study, the analysis of the expression patterns of melon LBD family genes revealed that genes in the Ic subclass (*CmLBD9*) and in the Ⅱb subclass (*CmLBD8*, *CmLBD20*, and *CmLBD30*) are highly expressed in roots, stems, leaves, flowers, fruits, and seeds, suggesting that they are involved in the development of various melon organs. In *Carya illinoensis*, *LBD30* and *LBD3* belong to Group II, and *LBD37* belongs to Group I; these genes are not only related to nutrient accumulation in the embryo, but are also involved in the processes of cell division and organ differentiation [27]. In *Capsicum annuum*, *Ca02g002988* has a higher expression level in roots than in other organs, while *Capana00g003460*, *Capana01g001405*, and *Capana06g000315* are all expressed only in roots. These genes may have a certain association with root morphogenesis [28].

Like many transcription factors, the LBD gene family forms a variety of molecular regulatory networks, playing important roles in plant growth and development and responses to environmental stress [29]. Multiple cis-acting elements in the gene promoter region play a crucial role in signal transduction, and their synergistic interactions can regulate complex biological processes. *AtLBD16* and *AtLBD29* are involved in growth hormone response and lateral root formation [29,30]. *AtLBD20* plays a role in the plant disease resistance process, mediated by the jasmonic acid signaling pathway [31]. Research has found that the LBD promoter contains many elements related to hormone regulation pathways, including abscisic acid, MeJA, GA, and IAA. MeJA is a plant hormone that regulates defense mechanisms and stress responses [32], while ABA is an important plant stress hormone that plays a crucial role in the salt stress signaling pathway [33]. Meanwhile, the LBD promoter also contains the base sequences of light response, stress response, and MYB specific binding sites. These different cis-acting elements together regulate the growth and development of melon, allowing it to adapt to unfavorable living environments and grow healthily.

LBD genes play an important role in plant defense responses. In the *Arabidopsis* LBD family, *LBD20* is a *F. oxysporum* susceptibility gene, marking the first demonstration of a LBD gene family member’s role in biotic stress [31]. Transcriptomic data of cassava under biotic and abiotic stresses showed that *MeASLBD46* and *MeASLBD47* were more responsive to disease and drought, and that *MeASLBD47* significantly reduced the virulence of cassava bacterial wilt (XamCHN11), according to real-time fluorescence quantitative reverse transcription PCR (qRT-PCR) and virus-induced gene silencing (VIGS) [34].

This study shows that after melon seedlings were inoculated with wilt bacteria, the expression levels of nine out of ten genes were significantly different in the stem. The significant upregulation of several LBD genes in stems suggests their involvement in the activation of defense pathways in response to pathogen invasion. After root infection by the wilt pathogen, mycelium forms and moves upward through the vascular bundle to invade the plant body. In the pathogenic mechanism of wilt disease, there are two main theories: the vascular occlusion hypothesis, and the toxin hypothesis. The vascular occlusion hypothesis believes that the blockage of xylem vessels by the pathogen is the main reason for plant wilting [35]. Our study found that compared to roots and leaves, the gene response in the stem was more intense, which may be related to the well-developed vascular bundles (xylem) in the stem, and the characteristic of wilt pathogens mainly damaging the vascular bundles (xylem). The dynamic regulation of LBD genes in the stem, reaching peak expression levels at 48 h and 96 h after inoculation, may indicate their key role in the early stages of plant-pathogen interaction. Moreover, compared to the stem, the expression patterns of LBD genes in roots and leaves showed significant differences. These results suggest that the response of melon LBD genes to wilt disease stress is tissue-specific, highlighting the complexity of LBD gene regulation in different plant organs.

Lü et al. analyzed the transcriptional situation of the non-host interaction between watermelon and *F. oxysporum*, finding that most genes involved in jasmonic acid (JA) biosynthesis were continuously upregulated, indicating that JA biosynthesis, along with the tryptophan-phenylalanine-lignin pathway, might play an important role in watermelon’s resistance against wilt pathogen infection [36]. Ding showed that black-seeded pumpkin primarily responds to wilt pathogen infection through disease resistance pathways mediated by ABA and JA [14]. Research by Zhou and Wu suggests that ABA signaling may play a significant role in cucumber’s defense response to *F. oxysporum* [37]. Studies on the gene expression profile of bananas at different infection times by wilt pathogen [38] conjecture that the infection by wilt pathogen in bananas is closely related to the JA signaling pathway.

In *Arabidopsis*, *AtLBD20* is considered to be a susceptible gene for *F. oxysporum* disease, and it is involved in the plant disease response mediated by jasmonate [39]. Cis-acting element analysis showed that *CmLBD2*, *CmLBD8*, *CmLBD14*, *CmLBD19*, and *CmLBD26* all contain methyl jasmonate (MeJA) responsiveness (CGTCA-motif); hence, it is inferred that these genes participate in the plant disease signaling pathway mediated by jasmonic acid methyl ester (MeJA). However, *CmLBD20* and *CmLBD30* do not contain CGTCA-motif, but both contain salicylic acid response elements (TCA-element), suggesting that these two genes resist wilt bacteria by participating in the signaling pathway mediated by salicylic acid (SA).

Our data indicate significant variations in the expression of the *CmLBD* gene at three selected time points post-inoculation (24, 48, and 96 h), providing crucial insights into the response of the melon LBD family to *Fusarium* infection. Nonetheless, we recognize that a more frequent analysis of time points could unveil finer details of dynamic expression patterns, which are of paramount importance for a comprehensive understanding of the melon LBD family’s reaction to infection. Therefore, future studies could consider including a broader range of time points for analysis, especially shorter intervals within the first 24 h, to delve deeper into the interactions between the host and pathogen.

Despite this study offering significant insights into the functionality of the LBD gene family in melon, particularly in resisting *F. oxysporum* infection, the applicability of these findings to other stress conditions or pathogens remains unclear. Future research should aim to explore the functions of LBD genes under a variety of biotic and abiotic stress conditions. Such an understanding will contribute to a multifaceted view of the roles of LBD genes in stress physiology and potentially enhance our capability to improve melon resistance through molecular breeding. Such investigations will require the integrated application of genomics, transcriptomics, and proteomics, among other approaches, to uncover the complex regulatory networks involving LBD genes.

## 5. Conclusions

At present, research on LBD genes of other species continues to emerge, which will help people further understand the LBD gene family. In this article, systematic analyses of the physical and chemical properties of LBD genes, chromosome positioning, collinearity, evolutionary tree, and cis-acting elements were carried out in sequence, aiming to provide a reference for more in-depth future research on the LBD gene family. In the interim, our study provides comprehensive insights into the expression profiles of melon LBD family genes in response to the wilt disease pathogen *F. oxysporum*. Nine genes exhibited strong expression in roots, stems, and leaves in response to pathogenic invasion. With the infection of *Fusarium*, the expression levels of these genes generally showed an increasing trend, among which *CmLBD20* and *CmLBD30* responded most intensely. It is speculated that these genes play significant biological roles during the *Fusarium* infection process. These results not only powerfully demonstrate that LBDs are involved in the melon’s resistance to biotic stress (*Fusarium* infection) but also, by analyzing the expression patterns of these ten CmLBD genes under *Fusarium* infection, our results provide robust molecular evidence for research into melon resistance breeding. Especially, the marked response exhibited by the *CmLBD20* and *CmLBD30* genes suggests they may play key roles in plant defense reactions, offering potential candidate genes for future molecular breeding efforts to improve melon’s resistance to *Fusarium* wilt. Our study lays a foundation for further understanding the function of the LBD gene family in the biology and pathology of melon plants and for the development of new molecular markers and creation of melon varieties with high resistance to *Fusarium* wilt. Overall, this research provides valuable resources and new strategic directions for improving melon quality and enhancing crop resistance to *Fusarium* wilt.

## Figures and Tables

**Table 1:** Information regarding the LBD family in melon.

Gene Name	Gene Symbol	Protein Length (Amino acids)	Molecular Weight (Da)	Isoelectric Point	Instability Index	Fat Solubility Index	Mean Hydrophilic Value	Subcellular Localization
*CmLBD1*	MELO3C000068P1	115	11,743.48	9.06	55.11	76.43	0.163	Nucleus
*CmLBD2*	MELO3C000076P1	99	10,950.40	5.67	61.24	69.90	−0.268	Nucleus
*CmLBD3*	MELO3C002290P1	167	18,814.35	8.52	47.79	73.05	−0.359	Nucleus
*CmLBD4*	MELO3C005009P1	204	22,340.62	6.41	72.35	91.86	−0.131	Nucleus
*CmLBD5*	MELO3C005013P1	177	20,037.16	7.60	62.04	82.20	−0.265	Nucleus
*CmLBD6*	MELO3C006183P1	231	24,953.60	8.93	80.51	80.69	−0.216	Nucleus
*CmLBD7*	MELO3C007098P1	240	27,293.82	7.62	74.98	64.62	−0.634	Nucleus
*CmLBD8*	MELO3C007620P1	232	25,560.77	7.58	66.53	69.40	−0.390	Nucleus
*CmLBD9*	MELO3C008309P1	172	19,111.61	8.10	62.87	68.72	−0.322	Nucleus
*CmLBD10*	MELO3C008779P1	201	22,171.17	8.96	54.69	70.90	−0.392	Nucleus
*CmLBD11*	MELO3C009989P1	177	19,508.68	7.58	68.27	54.63	−0.640	Nucleus
*CmLBD12*	MELO3C010752P1	193	21,546.77	7.99	51.03	88.13	−0.186	Nucleus
*CmLBD13*	MELO3C011135P1	96	10,508.77	4.75	31.24	87.60	−0.089	Nucleus
*CmLBD14*	MELO3C011730P1	173	19,126.14	6.56	79.98	82.37	−0.148	Nucleus
*CmLBD15*	MELO3C012250P1	217	23,478.48	8.45	36.69	72.40	−0.328	Nucleus
*CmLBD16*	MELO3C012624P1	160	17,234.61	8.14	72.61	70.19	−0.159	Nucleus
*CmLBD17*	MELO3C012741P1	184	20,243.07	6.28	68.12	80.22	0.014	Nucleus
*CmLBD18*	MELO3C012908P1	243	27,013.91	6.04	46.60	63.46	−0.500	Nucleus
*CmLBD19*	MELO3C016808P1	203	22,236.13	6.50	56.75	80.69	−0.202	Nucleus
*CmLBD20*	MELO3C017305P1	245	26,763.80	8.72	54.14	77.63	−0.240	Nucleus
*CmLBD21*	MELO3C018380P1	156	17,169.95	7.52	61.42	86.86	0.023	Nucleus
*CmLBD22*	MELO3C019314P1	238	26,823.12	8.88	46.86	67.94	−0.772	Nucleus
*CmLBD23*	MELO3C019995P1	222	23,851.07	9.10	79.33	75.63	−0.223	Nucleus
*CmLBD24*	MELO3C020121P1	244	26,813.14	8.61	76.41	70.00	−0.491	Nucleus
*CmLBD25*	MELO3C020944P1	310	34,948.92	7.12	67.35	64.87	−0.792	Nucleus
*CmLBD26*	MELO3C021578P1	165	18,202.35	7.66	50.92	66.30	−0.524	Nucleus
*CmLBD27*	MELO3C021789P1	170	18,490.96	8.32	62.51	73.47	−0.281	Nucleus
*CmLBD28*	MELO3C021964P1	265	29,996.60	5.00	55.56	75.81	−0.570	Nucleus
*CmLBD29*	MELO3C022505P1	228	24,793.47	6.97	47.74	80.44	−0.145	Nucleus
*CmLBD30*	MELO3C023802P1	302	32,712.42	8.86	51.46	82.35	−0.272	Nucleus
*CmLBD31*	MELO3C023880P1	221	24,271.06	4.45	48.49	78.10	−0.051	Nucleus
*CmLBD32*	MELO3C024038P1	236	25,568.78	7.06	45.07	75.34	−0.250	Nucleus
*CmLBD33*	MELO3C024387P1	288	32,397.20	5.42	51.16	76.60	−0.544	Nucleus
*CmLBD34*	MELO3C025504P1	102	11,653.77	9.43	52.87	76.47	−0.141	Nucleus
*CmLBD35*	MELO3C025701P1	161	18,044.50	4.94	56.38	73.35	−0.207	Nucleus
*CmLBD36*	MELO3C025742P1	198	21,505.30	5.59	75.26	81.36	−0.182	Nucleus
*CmLBD37*	MELO3C026269P1	185	20,363.90	7.63	53.81	71.30	−0.405	Nucleus

## Data Availability

Transcriptome data are available through the Melonet DB (https://melonet-db.dna.affrc.g-o.jp/ap/top).
